# CRISPLD2 Is Expressed at Low Levels during Septic Shock and Is Associated with Procalcitonin

**DOI:** 10.1371/journal.pone.0065743

**Published:** 2013-06-14

**Authors:** Tao Wang, Zhi-qin Wang, Lv Wang, Li Yan, Jian Wan, Sheng Zhang, Hong-quan Jiang, Wen-fang Li, Zhao-fen Lin

**Affiliations:** 1 Department of Emergency and Intensive Care Unit, Hainan Branch of Chinese PLA General Hospital, Sanya, Hainan Province, China; 2 Department of Emergency and Critical Care Medicine, Changzheng Hospital, Second Military Medical University, Shanghai, China; 3 Shanghai South Gene Technology Co. Ltd., Shanghai, China; D'or Institute of Research and Education, Brazil

## Abstract

**Introduction:**

Previous studies have shown that cysteine-rich secretory protein containing LCCL domain 2 (CRISPLD2) is a novel lipopolysaccharide (LPS)-binding protein, and the upregulation of CRISPLD2 expression protects mice against LPS-induced lethality. The aim of this study was to examine the expression of CRISPLD2 in patients with sepsis and characterize the association of this protein with procalcitonin.

**Methods:**

The expression of CRISPLD2 was determined in100 healthy volunteers and 119 septic patients. According to the definition of sepsis, patients were divided into three groups sepsis, severe sepsis, and septic shock. The relationship between CRISPLD2 levels and procalcitonin was also examined and statistically analyzed.

**Results:**

The CRISPLD2 levels in healthy individuals were 219.3±69.1 µg/ml. Patients with sepsis exhibited higher CRISPLD2 levels than observed in healthy individuals (p = 0.001), but CRISPLD2 expression was not upregulated in patients with septic shock. No significant differences were observed between the levels of CRISPLD2 in surviving and non-surviving spesis patients. CRISPLD2 levels were negatively correlated with procalcitonin levels(r = −0.334, p<0.001).

**Conclusions:**

The present study is the first to demonstrate the decreased expression of CRISPLD2 in septic shock and its association with PCT in sepsis. Further studies are needed to clarify the potential association between CRISPLD2 expression and clinical outcomes to determine if it could be used as a novel sepsis biomarker.

## Introduction

Sepsis is a major cause of death for critically ill patients in China, the USA and many European countries [1]–[3]. Lipopolysaccharide, a bacterial endotoxin, is a component of the outer membrane of Gram-negative bacteria that plays a key role in the pathogenesis of sepsis [4]–[8]. The principal plasma protein responsible for transporting endotoxin to immune effector cells is the lipopolysaccharide-binding protein (LBP) [9]–[11]. Assays designed to detect LBP are currently used to identify patients suffering from sepsis, but utilizing LBP as a sepsis biomarker has generated conflicting results [12]–[14].

Our previous work showed that the cysteine-rich secretory protein containing LCCL domain 2 (CRISPLD2/Crispld2) also functions as a LPS-binding protein with significant LPS-binding affinity [15]. We observed that the affinity of CRISPLD2 for LPS is similar to CD14 and MD2, higher than TLR4, but lower than LBP for LPS [15]. The stimulation of peripheral blood mononuclear cells (PBMCs) with LPS can induce the secretion of CRISPLD2 in vitro and in vivo. Treatment with recombinant CRISPLD2 inhibits the binding of LPS to target cells, reduces LPS-induced TNF-α and IL-6 production in vitro, and protects mice from endotoxin shock [15]. In our previous work, we also determined the concentration of CRISPLD2 in serum (or plasma) using immunoblotting assays and observed that the CRISPLD2 levels were significantly higher than that of LBP, soluble CD14 and others [15]. Notably, the CRISPLD2 levels in infant umbilical cord blood plasma were significantly lower than the CRISPLD2 concentration detected in adults [15]. These data implicates CRISPLD2 in the pathogenesis of sepsis. Procalcitonin (PCT) is widely used as a reliable marker for diagnosing and providing an accurate prognosis for sepsis [16]–[18].

Thus, the objective of this study was to examine whether CRISPLD2 serum levels are associated with PCT concentration in septic patients and disease severity. If this is correct, then CRISPLD2 in serum levels could be used as a biomarker to predict the clinical outcome of septic patients.

## Materials and Methods

### Patients

This study involved the prospective observation of a cohort of patients admitted to the emergency and intensive care units (15 ICU beds) of an urban university teaching hospital (928 beds total). The ethics committee of Changzheng Hospital in Shanghai, China approved this study. Written informed consent was obtained from the patients or a designated surrogate prior to enrollment in the study.

Trained research assistants screened the patients using the hospital’s electronic medical record system. All patients who had been diagnosed with sepsis, severe sepsis, and septic shock were considered for enrollment. Sepsis, severe sepsis, and septic shock were defined according to internationally accepted criteria [19]–[20]. The exclusion criteria included presence of infection with human immunodeficiency virus and/or AIDS, neutropenia not attributable to sepsis, treatment with immunosuppressive therapies (use of systemic glucocorticoids equivalent to 0.5 mg or more of prednisone/kg/day within a month prior to admission to the ICU; treatment with other major immunosuppressive drugs; or radiation therapy), pregnancy, or blood diseases (such as hematological tumors). Any patients refusing participation or whose relatives/representatives did not provide consent were also excluded.

Healthy individuals from the Health examination center affiliated with Changzheng hospital in Shanghai were enrolled and used in the control group, with an age criteria of >18 years and the patient must not have any acute medical or surgical conditions, that could potentially require admission to a hospital facility. The individuals in the control group who had been diagnosed with or showed evidence of any significant comorbid conditions were automatically excluded.

### Data Collection

Pertinent patient demographics including age, gender, comorbidities, site of infection, microbial isolates and major laboratory test results were recorded at baseline. All of the participants’ vital signs were measured and recorded at the time of enrollment. The disease severity in each patient was assessed at admission using two different scores: the acute physiology and chronic health evaluation (APACHE) II score [21] and the sequential organ failure assessment (SOFA) score [22]. The comorbidities were measured using the Charlson Comorbidity Index (CCI) [23]. The CCI is a weighted 19-item index, and the total score is calculated as the sum of the weights of each comorbid condition presented in each patient [23].

### Primary Data Analysis

We collected blood serum samples within 24 hours after admission. The serum was transferred to plastic tubes and stored at −80°C until further use. The procalcitonin levels were measured using an immunoluminometric assay (LUMItest PCT kit, BRAHMS Diagnostica, Hennigsdorf bei Berlin, Germany). The chemiluminescence was measured using a luminometer (Lumat, LB 9507, Berthold, Wildbad, Germany). CRISPLD2 in serum was measured by competitive ELISA as described in a previous study [15].

### Statistics

Data analysis was conducted using SPSS 18 software. The data are presented as the means±SD, median and interquartile ranges (IQR, 25th and 75th percentiles) or numbers and percentages. The categorical variables were compared using Chi-square or Fisher’s exact tests, where appropriate.We calculated the differences in parametric data between different strata, using Student’s t test and ANOVA with post hoc LSD test for the two groups. For comparisons of the nonparametric data, the Mann-Whitney test was used for the two groups, and the Kruskal-Wallis test with post hoc Mann-Whitney test was performed for multiple comparisons. Spearman’s rank correlation or Pearson’s tests were performed to evaluate the association between the two groups, where appropriate. We analyzed the data using the receiver operating characteristic (ROC) curve for serum CRISPLD2, PCT levels at admission, and for the 28-day mortality prediction. In all analyses, p<0.05 was considered significant.

## Results

### Characteristics of the Sepsis Patients and Healthy Individuals in the Control Group

Patients admitted to the ICU were screened for this study. A total of 380 patients were screened, and 160 did not meet inclusion criteria, and were excluded (without appropriate samples (n = 32) and declining to participate (n = 69)). Subsequently, 119 patients were analyzed in the present study.

A summary of the patient demographics and clinical parameters of the study population arelisted in Table 1. In total, 119 sepsis patients and 100 healthy volunteers met the inclusion criteria. The mean age of the sepsis patients was significantly higher than that of the healthy volunteers (50.3 (18.5) *versus* (vs) 46.0 (17.3) years, p = 0.001). Males represented 76.4% of the cohort of patients and 58% ofthe healthy volunteers (p = 0.001). The primary sources for sepsis infection were pneumonia, and abdominal infections, with a predominance of gram-negative bacterial infections. The mortality rate was 26.1% for all patients with a higher mortality for severe sepsis and septic shock.

**Table 1:** Patient characteristics based upon the diagnosis at admission.

	Healthy	Sepsis	Severe sepsis	Septic shock	P value*
**N**	100	54	49	16	
**Male (%)**	58 (58)	38 (70.4)	39 (79.6)	11 (68.9)	0.498
**Age (sd)**	46.0 (17.3)	49.8 (17.9)	46.1 (18.7)	64.6 (12.7)	0.002
**APACHEII^ ad^ (sd)**		11.0 (5.8)	17.7 (5.8)	23.1 (5.4)	0.000
**SOFA^ ad^ (sd)**		3.5 (2.6)	7.3 (3.2)	12.0 (3.5)	0.000
**Infection site**					
** Respiratory (%)**		21 (38.9)	18 (36.7)	9 (56.3)	0.369
** Abdominal (%)**		9 (16.7)	20 (40.8)	6 (37.5)	0.020
** Others (%)**		24 (44.4)	11 (22.4)	1 (6.3)	0.004
**Comorbidities**					0.007
** CCI = 0 (%)**		35 (64.8)	16 (67.3)	4 (25.0)	
** CCI≥1 (%)**		19 (35.2)	16 (32.7)	12 (75.0)	
**ICU mortality (%)**		5 (9.3)	14 (28.6)	12 (75.0)	0.000
**PCT (ng/ml)**	0.11 (0.07–0.15)	0.6 (0.3–1.4)	4.7 (1.7–13.2)	15.4 (3.7–49.6)	0.000
**WBC (** * **10^9^/l)**	6.32 (1.52)	11.7 (5.4)	12.7 (6.2)	13.6 (5.9)	0.514
**Albumin (g/l) (sd)**		34.7 (4.5)	31.7 (7.3)	32.1 (6.4)	0.437
**Creatine (µmol/L)**		79.1 (85.9)	129.9 (179.4)	140.7 (111.4)	0.018
**Platelet (** * **109/l)**		194.2 (98.1)	150.4 (105.1)	112.1 (75.8)	0.004
**TBIL (µmol/L)**		26.5 (25.1)	30.6 (35.7)	44.4 (39.8)	0.059
**Hematocrit (%)**		31.8 (6.7)	29.1 (7.9)	30.2 (5.6)	0.103
**CRISPLD2 (µg/ml)**	219.3(61.0)	247.2 (78.6)	238.2 (76.9)	142.0 (47.0)	0.000
**Microbial Isolates (%)**					
** Gram-positive**		5 (9.3)	4 (8.2)	0 (0)	0.459
** Gram-negative**		16 (29.6)	20 (40.8)	6 (37.5)	0.485
** Polymicrobial**		17 (31.5)	13 (26.5)	8 (50.0)	0.216
** Others**		16 (29.6)	12 (24.5)	2 (12.5)	0.299
** Coexistes with fungi**		8 (14.8)	5 (10.2)	4 (25.0)	0.336

ad: admission; sd: standard deviation; CCI: Charlson comorbidity weight index; WBC: white blood cells; CRISPLD2: cysteine-rich secretory protein containing LCCL domain 2; APACHEII, acute physiology and chronic health evaluation score; SOFA: sequential organ failure assessment score; TBIL: total bilirubin; PCT: procalcitonin; ICU: intensive care unit.

* Compared with sepsis, severe sepsis and septic shock patients.

The scores generated using APACHEII, SOFA, CCI, the platlet counts, and the creatine and PCT levels differed significantly among the three groups of sepsis patients. The white blood cell count, HCT percent, and total serum bilirubin levels were not significantly different.

### Distributions of Serum CRISPLD2 in Healthy People

**Figure 1:**
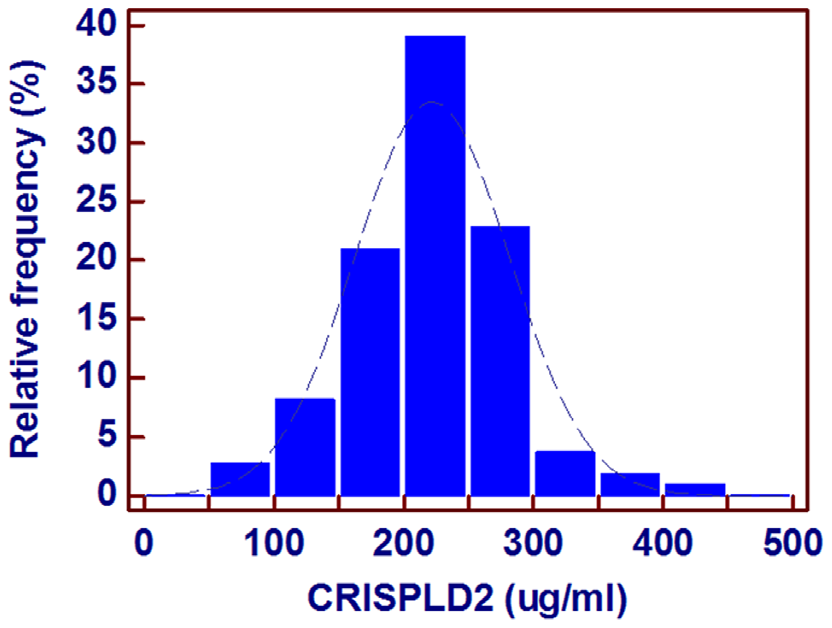
Distribution of serum CRISPLD2 concentration in healthy volunteers.

In the 100 healthy controls, the average CRISPLD2 level was 219.3±61.0 µg/ml; the minimum value was 55.9 µg/mland the maximum was 437.2 µg/ml. The distribution of serum CRISPLD2 is presented in Figure 1. The PCT levels (median (IQR) 0.11 ng/ml (0.07–0.15 ng/ml)) and WBC counts (6.32±1.52 *10^12^/L) were also determined. The CRISPLD2 level was not significantly associated with PCT, WBC, age or sex in healthy population.

### Serum Level of CRISPLD2 and PCT in Patients with Different Statuses at Admission

**Figure 2:**
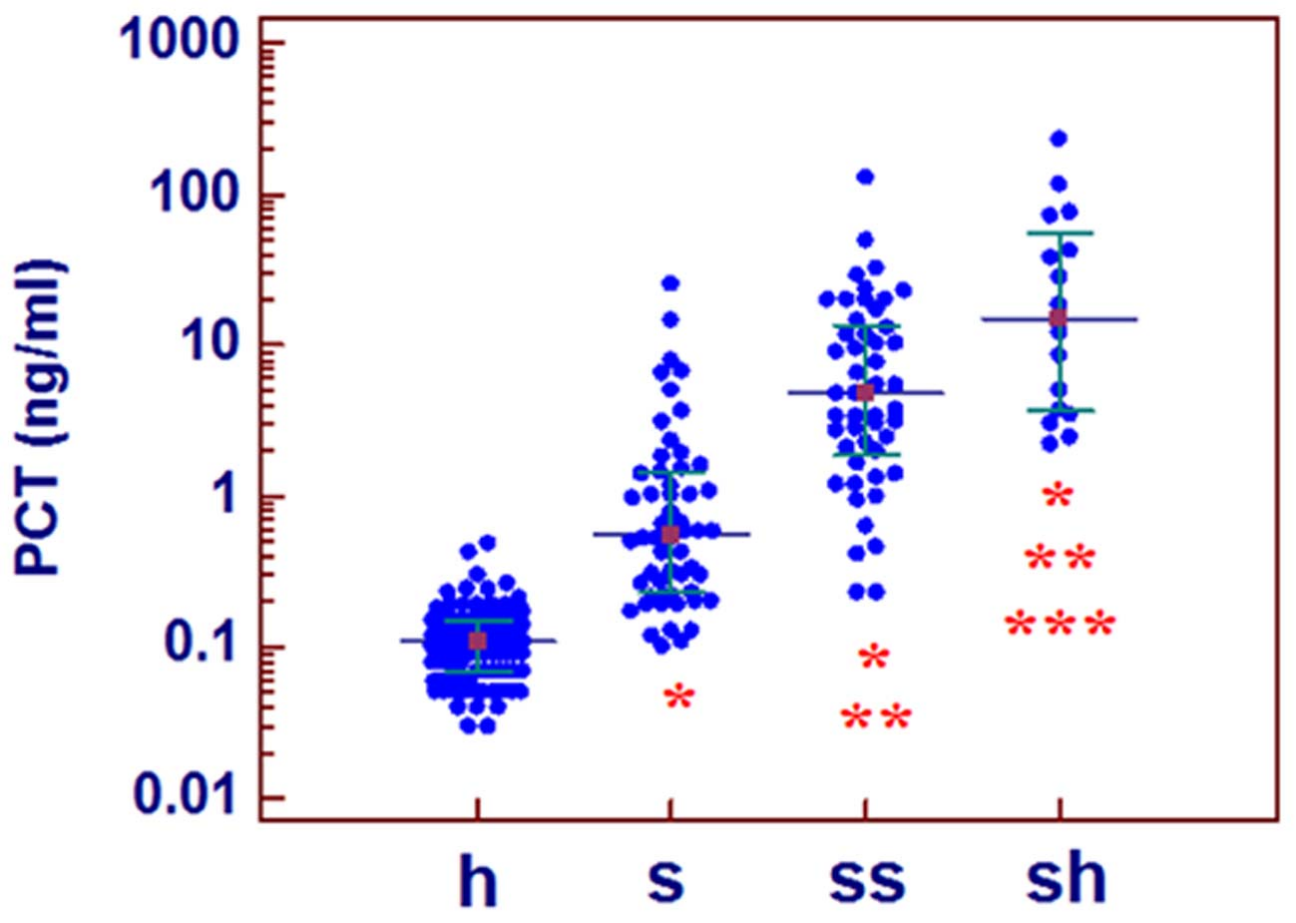
Comparison of the PCT levels in healthy controls and sepsis patients. (p<0.05: *compared with h, **compared with s, ***compared with ss.); h: healthy controls; s: sepsis; ss: severe sepsis; sh: septic shock.

**Figure 3:**
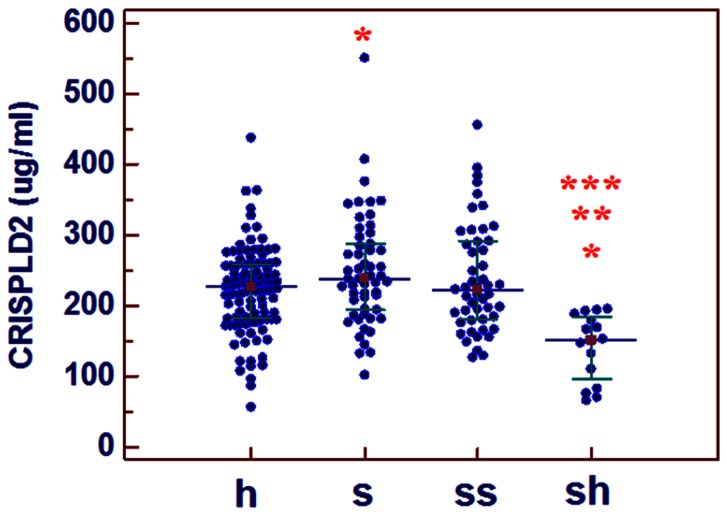
Comparison of the CRISPLD2 levels in healthy controls and sepsis patients. (p<0.05: *compared with h, **compared with s, ***compared with ss.); h: healthy controls; s: sepsis; ss: severe sepsis; sh: septic shock.

At admission, the PCT concentration differed significantly among four groups: healthy, sepsis, severe sepsis, and septic shock. The CRISPLD2 level was significantly higher in sepsis patients compared with that in the healthy population (p = 0.019) and significantly lower in patients with septic shock compared with that in healthy, sepsis, and severe sepsis (all p<0.001). The CRISPLD2 concentration was not significantly different between severe sepsis and healthy (p = 0.161), and no significant difference was observed between severe sepsis and sepsis patients (p = 0.445). (Figure 2 and Figure 3).

The age, sex, mechanical ventilation, and comorbidity (indicated by CCI) had no effect on PCT and CRISPLD2 concentrations in patients. Patients with abdominal infections had higher PCT and lower CRISPLD2 levels than patients with respiratory infections. Notably, more patients experienced severe sepsis and septic shock through abdominal infections. The PCT concentration was higher in patients with gram-negative isolates than in other patients. The CRISPLD2 level was slightly lower in patients with gram-negative isolates, but there was no statistical significance. (Table 2).

**Table 2:** CRISPLD2 and PCT serum levels with different statuses in patients.

	n	PCT (ng/ml)	P value	CRISPLD2 (*µ*g/ml)	P value
**Sex**					
** Male**	88	2.3 (0.5–10.4)	0.509	235.4 (83.0)	0.214
** Female**	31	1.5 (0.6–5.5)		214.2 (77.1)	
**Age (years)**					
** ≤44**	48	2.2 (0.6–11.2)	0.992	225.6 (78.6)	0.311
** 45–65**	36	3.1 (0.5–7.9)		246.8 (82.7)	
** ≥65**	35	1.6 (0.5–5.8)		218.4 (84.5)	
**Infection sites**					
** Respiratory**	48	1.4 (0.5–3.2)	0.001	251.7 (100.2)	0.056
** Abdominal**	35	6.6 (2.4–19.9)		214.6 (68.2)	
** Others**	36	1.1 (0.3–5.2)		215.7 (58.6)	
**Comorbidities**					
** CCI = 0**	72	2.3 (0.5–7.8)	0.672	232.9 (79.2)	0.624
** CCI≥1**	47	2.1 (0.6–13.3)		225.3 (86.2)	
**Mechanical ventilation**					
** Yes**	48	2.2 (0.6–8.1)	0.859	236.0 (90.9)	0.508
** No**	71	2.4 (0.5–10.1)		225.8 (75.3)	
**Progonosis**					
** Survivors**	88	1.4 (0.5–5.4)	0.003	231.3 (82.6)	0.754
** Non-survivors**	31	5.5 (2.2–19.4)		225.9 (80.3)	
**Microbial Isolates**					
** Gram-positive only**	9	1.2 (1.0–3.4)	0.011	236.7 (92.1)	0.294
** Gram-negative only**	42	4.3 (2.3–10.1)		218.0 (83.8)	
** Others (fungi or mxied)**	68	1.4 (0.4–5.8)		236.3 (79.5)	

PCT: procalcitonin; CCI: Charlson comorbidity weight index.

### The Association of the Serum CRISPLD2 Levels with Other Variables

**Figure 4:**
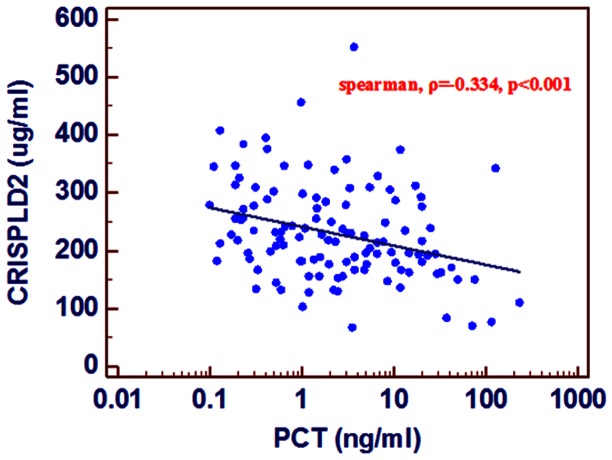
Correlation between the levels of PCTand CRISPLD2 in sepsis patients.

There was a significant negative correlation between the CRISPLD2 serum concentration and the PCT level (Spearman, ρ = −0.334, p<0.001)(Figure 4), SOFA score (Pearson, r = −0.201, p = 0.028, white blood cell count (Pearson, r = −0.229, p = 0.014), GCS score (Pearson, r = 0.195, p = 0.035), and creatine level (Pearson, r = −0.183, p = 0.051). There was no significant difference in the APACHEII scores, (Pearson, r = −0.115, p = 0.212), age (Pearson, r = 0.033, p = 0.720), hematocrit (Pearson, r = 0.085, p = 0.364), serum albumin level f (Pearson, r = 0.054, p = 0.552), PLT (Pearson, r = 0.055, p = 0.555), and TBIL (Pearson, r = −0.123, p = 0.188).

### Serum CRISPLD2 Level Predict 28-day Mortality

The PCT concentration was significantly higher in non-survivors than in survivors, but the CRISPLD2 serum level did not significantly differ between the two groups (Table 2). The areas under the ROC curve for PCT and CRISPLD2 that were used to predict 28-day mortality were 0.680 (95% CI: 0.588 to 0.763) and 0.519 (95% CI: 0.425 to 0.611).

## Discussion

To our knowledge, this is the first study concerning the levels of CRISPLD2 in human subjects with sepsis. Here, we conducted a prospective observational study, and found a considerable fluctuation in the CRISPLD2 concentrations of healthy populations compared with sepsis patients. Patients with mild sepsis had slightly higher levels of CRISPLD2 than healthyindividuals. Patients with septic shock exhibited lower levels of CRISPLD2 than healthy, sepsis, and severe sepsis patients. Furthermore, we also observed a statistically significant inverse relationship between serum CRISPLD2 and PCT levels, but serum CRISPLD2 levels between survivors and non-survivors did not significantly differ.

CRISPLD2 plays a role in the development of rat lung and mouse kidney and is also involved in the development of nonsyndromic cleft lip with or without cleft palate [24]–[28]. A recent study [15] demonstrated that CRISPLD2 is a novel LPS-binding protein expressed in multiple tissues and leukocytes. Human peripheral blood granulocytes and mononuclear cells including monocytes, NK cells, and T cells, spontaneously release CRISPLD2. CRISPLD2 exhibits significant LPS-binding affinity similar to that of soluble CD14, prevents LPS binding to target cells, reduces LPS-induced TNF-α and IL-6 production, and protects mice against endotoxin shock. The upregulation of CRISPLD2 through pretreatment with LPS or the exogenous expression of recombined CRISPLD2 protein protects mice against LPS lethality [15].

The data obtained in the present study showed that patients infected with gram-negative bacterial isolates showed a lower level of CRISPLD2 than those with gram-positive bacterial infections. Indeed, CRISPLD2 is not only a LPS-binding protein, but also a single-stranded nucleic acids (ssDNA)-binding protein. Recently, the RAAIH (R3H)-domain, which binds ssDNA, was identified in many mammalian cysteine-rich secretory proteins [29]. We have previously shown that CRISPLD2 contains an R3H structure and is also a DNA- binding protein (unpublished data). Notably, treatment with ssCpG-DNA protects bacterial-infected animals [30]–[32], but it is unknown whether CRISPLD2 modulates ssCpG-DNA-induced protective responses. We also observed that PAMPs, such as peptidoglycan, ssDNA, and virus Poly I: C and DAMPs, such as HMGB1, stimulate PBMCs or THP-1 cells to secrete CRISPLD2 (unpublished data). These data implicate CRISPLD2 not only in gram-negative bacterial but also polymicrobial sepsis.

The results obtained in the present study are consistent with those of previous work [15], showing that the CRISPLD2 serum levels were significantly different in sepsis patients compared with the healthy control group. The changes in CRISPLD2 expression might reflect the host communicating with the sensed microbial environment. In a previous study, they found reduced CRISPLD2 concentration in infant umbilical cord blood plasma when compared to thatmeasured in healthy adults [15]. We also observed prolonged treatment with broad-spectrum antibiotics downregulates serum Crispld2 levels in mice (unpublished data). In addition, the CRISPLD2 levels in sepsis patients and healthy controls were similar, and, unlike PCT, the expression of CRISPLD2 did not change with the increasing severity of disease. The CRISPLD2 level might potentially reflect the host’s response to infection, where the failure to upregulate CRISPLD2 expression indicates that the infection has entered a severe stage and/or is out of control (such as is observed in patients with septic shock or high PCT levels).

Interestingly, low CRISPLD2 concentrations were observed in septic shock. The lower level of CRISPLD2 expression during septic shock might indicate increased utilization or reduced production of this protein. However, the observational nature of the present study makes it difficult to determine which factor plays a major role in sepsis development. Patients with septic shock could consume more CRISPLD2 as a result of persistent bacterial antigen stimulation. A limited ability to produce CRISPLD2 might also contribute to the low protein level observed during septic shock. Many studies have shown that immunosuppressed patients exhibit severe sepsis and septic shock [33]–[35]. Boomer observed that sepsis patients that develop an immunosuppressed status do not survive and show a significant reduction of the cytokine levels in splenocytes after LPS or anti-CD3/anti-CD28–stimulation in vitro compared with non-sepsis patients [36]. Whether low CRISPLD2 levels in sepsis patients reflects immunosuppression remains elusive.

The results obtained in the present study did not show significant differences in the CRISPLD2 levels between survivors and non-survivors. A previous study [15]showed that the levels of CRISPLD2 was associated with lethal doses of LPS in a mouse endotoxin-shock model and upregulating the level of CRISPLD2 expression reduced mortality. Unfortunately, animal models used in sepsis studies do not always reflect applicable clinical facts. Whether treating patients suffering from severe sepsis with recombinant CRISPLD2 protein, or other strategies, halts or reverses progression of the disease remains unknown.It would be interesting to monitor CRISPLD2 serum levels in patients with increased risk of developing septic shock while actively inducing a boost in CRISPLD2 concentration once the levels of this protein fall below a critical threshold.

PCT is a reliable marker to diagnosis and differentiate sepsis, severe sepsis, and septic shock. Within populations of patients affected with sepsis, an elevation of the PCT levels has been associated with poor outcomes [16]–[18]. In fact, a statistically significant association between CRISPLD2 and PCT levels in sepsis patients was demonstrated in the current study. We also observed that the CRISPLD2 level was negatively associated with the SOFA and GCS scores, the white blood cell count and/or the creatine level. Although the PLT count and the TBIL level differ significantly in patients with different sepsis severities, there was no correlation with the CRISPLD2 levels. In addition, age, sex, comorbidities, sources and sites of infection did not affect the CRISPLD2 level.

LBP is a major LPS-binding protein in the mammalian biological system, which facilitates the transfer of LPS to the membrane-bound CD14 receptor and then induces inflammation at local sites of infection [37]–[39]. LBP has been proposed as a sensitive marker for bacterial infection and a useful follow-up parameter in detection and resolution of sepsis. CRISPLD2 expression *in vivo* is different compared with LBP. LBP is constitutively expressed in human plasma at low concentrations (3–15 mg/ml) and can increases up to 200 mg/ml during the acute phase response 40–41. Notably, CRISPLD2 might be functionally opposite proteins, suggesting that CRISPLD2 effectively blocks LBP-mediated-LPS-induced immune functions, although the mechanism of this antagonism is unclear [15].

Glucocorticoids trigger the upregulation of CRISPLD2 expression, as glucocorticoid receptor-binding elements are present in the promoter region of CRISPLD2 [42]. Thereby, treatment with glucocorticoids not only reduces the production of pro-inflammatory cytokines in sepsis [43]–[45] but also simultaneously to up-regulates CRISPLD2 protein secretion.

Conclusions obtained from research presented here are restricted by the observational nature of this study and limited knowledge of CRISPLD2. First, the current study was not designed to determine which factors might directly or indirectly affect CRISPLD2 concentration in patients. Second, the dynamic observation of CRISPLD2 levels in patients might offer more informations concerning severity and mortality than inferred by the simple determination of CRISPLD2 levels detected on admission in the present study. Third, we did not determine the serum levels of other endotoxin-binding proteins, endotoxins, or other inflammatory markers. Fourth, the LUMI test (Chemiluminescence) was used in this study to examine the PCT levels, which has a lower sensitivity compared with the current assays [46]–[47]. Finally, the control groups were younger and comprised more females than the sepsis patient groups. However, age and gender did not impact the CRISPLD2 levels in the healthy controls and sepsis patient groups included in the present study. These limitations prompt further research opportunities in the future.

The decreased expression of CRISPLD2 in septic shock and its association with PCT suggests CRISPLD2 as a potential biomarker in sepsis. Within the limitations of the study, the CRISPLD2 expression was not correlated with the outcome of patients. More research is needed to explore the function of CRISPLD2, the factors affecting the expression of this protein, and its clinical relevance.
